# Influence of arterial blood gases on the renal arterial resistive index in intensive care unit

**DOI:** 10.1186/s12967-023-04407-w

**Published:** 2023-08-12

**Authors:** Stéphanie Ruiz, Fanny Vardon-Bounes, Marie Virtos, Thierry Seguin, Laure Crognier, Antoine Rouget, Bernard Georges, Jean-Marie Conil, Vincent Minville

**Affiliations:** 1 Department of Anesthesiology and Intensive Care, Rangueil University Hospital, University Hospital of Toulouse, University Paul Sabatier, Avenue Jean Poulhès, Toulouse, France; 2 RESTORE, UMR 1301, Inserm CNRS-Université Paul Sabatier, Université de Toulouse, 5070 Toulouse, France

**Keywords:** Renal artery Doppler, Resistive index, Acute kidney injury, Arterial blood gases, Intensive care unit

## Abstract

**Background:**

Renal artery Doppler sonography with resistive index (RI) determination is a noninvasive, fast, and reliable diagnostic tool increasingly used in the intensive care unit (ICU) to predict and assess the reversibility of acute kidney injury (AKI). However, interpreting the RI can be challenging due to numerous influencing factors. While some studies have explored various confounding factors, arterial blood gases have received limited attention. Therefore, our study aims to evaluate the impact of arterial blood gases on the RI in the ICU setting.

**Methods:**

This prospective observational study enrolled ICU patients who required blood gas analysis and had not experienced significant hemodynamic changes recently. The RI was measured using standardized Doppler ultrasound within an hour of the arterial blood gases sampling and analysis.

**Results:**

A total of sixty-four patients were included in the analysis. Univariate analysis revealed a correlation between the RI and several variables, including PaCO_2_ (R = 0.270, p = 0.03), age (R = 0.574, p < 0.0001), diastolic arterial pressure (DAP) (R = − 0.368, p = 0.0028), and SaO_2_ (R = − 0.284, p = 0.0231). Multivariate analysis confirmed that age > 58 years and PaCO2 were significant factors influencing the RI, with respective odds ratios of 18.67 (p = 0.0003) and 1.132 (p = 0.0267).

**Conclusion:**

The interpretation of renal arterial RI should take into account thresholds for PaCO_2_, age, and diastolic arterial pressure. Further studies are needed to develop a comprehensive scoring system that incorporates all these cofactors for a reliable analysis of RI levels.

*Trial registration* This observational study, registered under number 70–0914, received approval from local Ethical Committee of Toulouse University Hospital.

## Introduction

Acute kidney injury (AKI) is a significant complication that affects approximately 30–40% of patients in the intensive care unit (ICU) and 1% of patients undergoing major postoperative surgery [1, 2]. It serves as an independent prognostic factor for mortality [1, 2]. Consideration of the consequences of AKI, particularly the need for dosage adjustments, is a matter of priority for the management of ICU patients [3]. Conversely, some patients (those with multiple trauma, severe burns or serious head injuries) exhibit augmented renal clearance (ARC), which also requires dosage adjustments to prevent inadequate drug dosing and its associated clinical or biological consequences [4].

The renal interlobar arterial resistive index (RI), initially introduced by Pourcelot for the study of renal perfusion, is increasingly utilized in various renal disorders, including graft dysfunction and obstructive nephropathies [5, 6]. This noninvasive and reproducible tool, conveniently applicable at the patient’s bedside, has proven its worth in early detection and prediction of the reversibility of AKI among ICU patients [7]. Additionally, this index is integrated into personalized management strategies for the hemodynamics of patients with septic shock [8–10]. An RI threshold of ≥ 0.7 is employed for AKI diagnosis, while more recently, other thresholds have been proposed to differentiate AKI presence in the context of sepsis or to predict the risk of persistent AKI [8, 9, 11, 12].

The relationship between renal macro hemodynamics and renal function is not linear. Multiple independent factors can influence the resistive index, including vascular resistance, vascular compliance, and mean arterial pressure [13]. Consequently, it is now recognized that atherosclerosis, diabetic nephropathy, primary hypertension, renal artery stenosis, and chronic kidney failure have an impact on the RI [14]. Additionally, hypoxemia and hypercapnia have been associated with renal arterial vasoconstriction [15, 16]. Previous studies, albeit with small sample sizes, have demonstrated the influence of arterial blood gases on the RI in patients with chronic respiratory insufficiency, kidney transplantation, and ICU patients with acute respiratory distress syndrome (ARDS) [16, 17].

Therefore, the primary objective of this study was to evaluate the influence of arterial blood gases on the RI in ICU patients.

## Materials and methods

### Patients

This prospective, observational study included adult patients admitted to the ICU. Patients who required arterial blood gas analysis and had not experienced changes in catecholamine administration within the past hour were eligible for inclusion. The inclusion process commenced at the time of blood gas analysis.

Exclusion criteria encompassed the following: individuals under 18 years of age, pregnant women, incapacitated adults, patients with a history of chronic kidney failure, kidney malformation, a single kidney, renal artery stenosis, or diabetic nephropathy. Patients who were undergoing dialysis or had received diuretic treatment within the past 4 h were also excluded. Furthermore, patients with hemodynamic failure necessitating an increased dosage of catecholamines in the hour before measurements, as well as those with therapeutic limitations, were not included in the study.

This observational study, registered under number 70–0914, received approval from local Ethical Committee of Toulouse University Hospitals. The study was performed according to the declaration of Helsinki. No change in our current clinical practice (measured creatinine clearance monitoring, at least once a week, is a part of the routine medical care of the patients) and no randomization was performed. As it was an observational study, in accordance with French law, oral informed consent was required.

### Protocol and data collection

At inclusion we collected the demographic data (age, sex, reason for admission in ICU, the severity scores [SAPS II, SOFA], weight, height and history), and the ventilator and hemodynamic clinical data (ventilatory mode, PEP, FiO_2_, tidal volume, expiratory volume, respiratory rate, heart rate and blood pressure). The blood gas and ultrasound data were collected over a period of less than one hour (PaO_2_, PaCO_2_, pH, HCO_3−_, blood content of O_2_ and CO_2_, RI). The other laboratory data were from daily assessments of the patient.

The renal Doppler was performed as previously described with a 4.5 MHz probe, with the patient in 30° supine position [18, 19]. Briefly, the kidneys and the interlobar arteries were located using B mode ultrasound and color Doppler in oblique longitudinal section via posterolateral approach. After an initial morphological study, the best-visualized kidney was selected for measurement of the velocities in inter lobar arteries in B-mode and pulsed wave Doppler. These arteries are located in the deep cortex in the middle portion of the kidney. The optimal gain was obtained from the velocity curves in pulsed wave Doppler. The peak systolic and end-diastolic velocities were measured. The RI was automatically calculated (RI = peak systolic velocity − end-diastolic velocity/peak systolic velocity). The RI was calculated with three to five successive measurements and the RIs were averaged [18–21]. An RI > 0.7 is described as pathological, indicative of obstructive kidney failure or renal parenchymal impairment.

### Statistical analysis

After a first descriptive statistical step and verification of the normal distribution of values (Kolmogorov–Smirnov test), the studied population was separated into three groups according to the glomerular filtration rate (decreased GFR for CrCl measured < 60 mL/min/1.73 m^2^; normal GFR for CrCl between 60 and 150 /160 mL/min/1.73 m^2^; ARC if CrCl > 150 (women) and 160 mL/min/1.73 m^2^ (men)) [22].

The patient characteristics between the different groups were compared using non-parametric tests (Mann–Whitney test and Kruskal–Wallis test) due to the non-homogeneity of the group sizes. The results are expressed as median and 95% confidence intervals (95% CI).

Then, we analyzed the relationship between the RI and the quantitative variables, including age, GFR, the hemodynamic parameters and those characterizing arterial blood gases (linear regression through Pearson’s r coefficient and/or Spearman’s rank correlation coefficient). Next, we integrated the significant variables in multiple regressions to describe a model that included the only factors of interest.

As the limited population of patients with an alteration in renal function did not enable calculation of a statistically valid RI threshold, a RI threshold > 0.7 was chosen since it is found in most published studies to date [18, 19]. The discriminant value of the covariates of interest based on the RI was assessed by studying the receiver operating characteristic (ROC) curves and their associated areas under the curve (AUC).

A multi-dimensional analysis through logistic regression evaluated the association between the covariates for which the p value was < 0.2 and the dependent variable (RI > 0.7) through measurement of the odds ratio. We tested several models by choosing that for which the observed data was best adjusted to the model (Hosmer-Lemeshow test, percentage of correctly classified cases, area under the curve of the model).

To highlight patients at risk of having an RI > 0.7, a partitioning of the population was represented using a Classification and Regression Trees (CART) analysis. The advantage of this approach is to describe the means of distribution of the population in homogeneous groups according to RI level and the covariates selected from the multidimensional analysis.

Statistical analysis was conducted using MedCalc® statistical software, version 15 (Mariakerke, Belgium) for the majority of the analyses except for the CART method, which was carried out on SPSS® for Window version 24 (IBM Corporation, Chicago, IL). A p value < 0.05 was considered statistically significant.

## Result

### Population

Among the 64 patients included in the study, the median interval between ICU admission and inclusion was 5 (3–7) days. 53% of the patients were under controlled mode ventilation, 30% were on non-invasive mode during the procedure, and 16% were on spontaneous ventilation. Table 1 presents the clinical, biological, and hemodynamic data. The included patients had a median age of 55.5 years (50–61) and a SAPS II score of 49 (95% CI: 44–55) upon admission.

**Table 1:** Clinical, biological and hemodynamic data

	Median	CI 95%	Min.	Max.
*Clinical data*				
Age (years)	55.5	50–61	17	87
Albuminemia (g/L)	23	21–26	14	36
BMI (kg/m^2^ )	25	24–27	17	37
SAPS 2	49	44–55	23	91
SOFA score	4	4–6	1	12
Delay between ICU admission and inclusion (days)	5	3–7	1	25
PEEP (cmH_2_O)	6	5–7	0	12
*Biological data*				
Plasma creatinine (µmol/L)	62.5	57–68	26	307
BUN (mmol/L)	6.8	5.7–7.5	1.9	51.9
CKD-EPI (mL/min/1.73 m²)	109	100–114	18.6	145
Measured creatinine clearance (mL/min/1.73 m²)	118	105–134	9	319
pH	7.43	7.42–7.44	7.24	7.55
PaO_2_ (mmHg)	82	75–96	41	191
SaO_2_ (%)	97	95.3–98	82	100
PaCO_2_ (mmHg)	37	35.7–40	27	66.5
Plasma total CO_2_ content (mmol/L)	25.5	24.9–26.4	17.4	40.9
Hemoglobin (g/dL)	10.1	9.6–10.5	7.8	13.6
*General hemodynamic data*				
SAP (mmHg)	130	126–136	90	177
DAP (mmHg)	65.5	61–69	42	98
MAP (mmHg)	85	83–88	59	120
Pulse pressure (mmHg)	66	59.7–69	29	106
Heart rate (bpm)	89	84–93	39	150
Renal hemodynamic data				
Pulsatility Index	1.33	1.1–1.5	0.89	2.1
Resistivity Index	0.69	0.66–0.72	0.48	0.86

*BMI* body mass index, *BUN* blood urea nitrogen, *CI* confidence interval, *DAP* diastolic arterial pressure, *ICU* intensive care unit, *MAP* mean arterial pressure, *PEEP* positive end-expiratory pressure, *SAP* systolic arterial pressure, *SAPS* Simplified Acute Physiology Score, *SOFA score* Sepsis-related Organ Failure Assessment score

### Univariate analysis

**Fig. 1:**
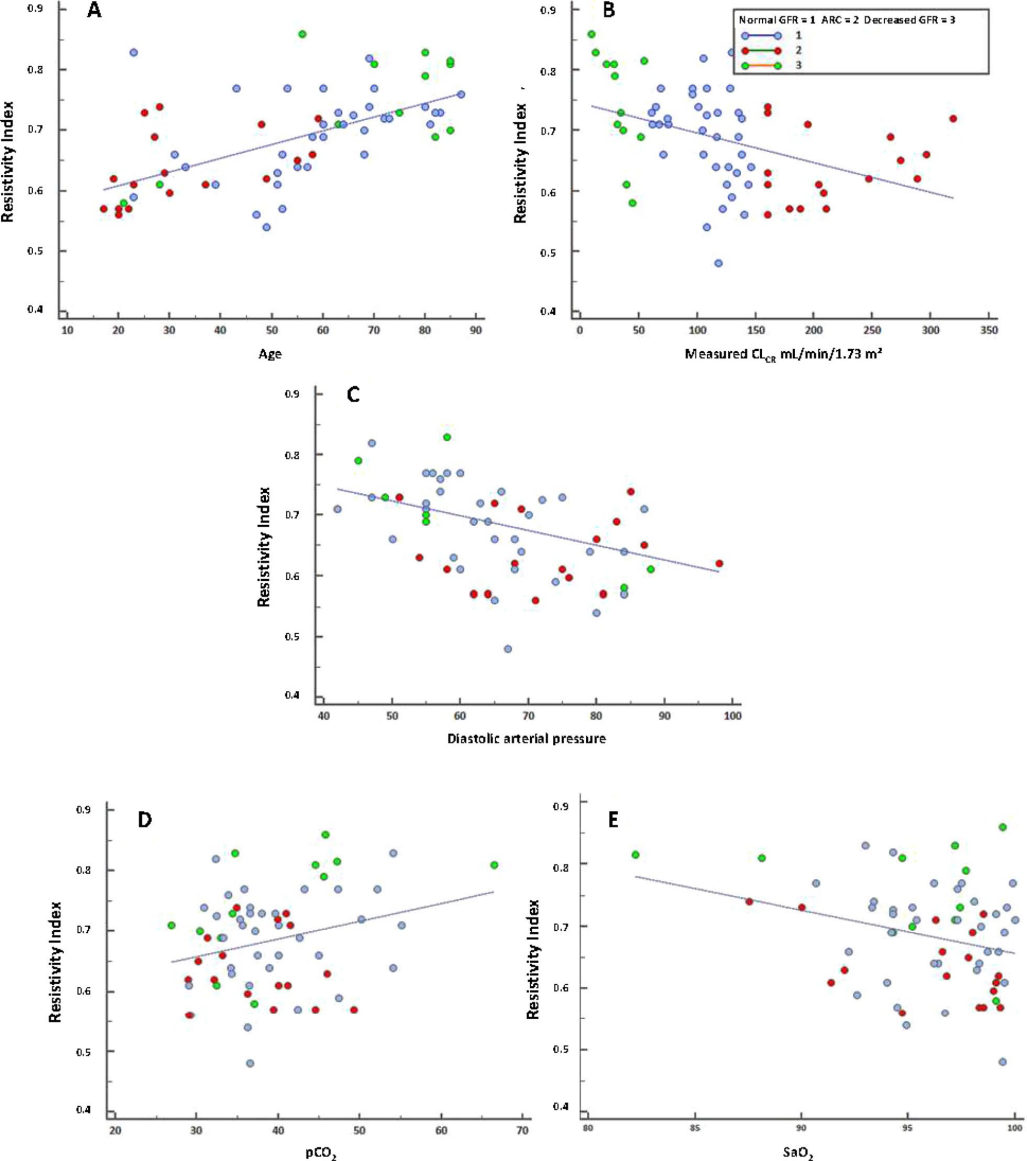
Relationship between resistivity index (RI), age measured creatinine clearance (CL_CR_), diastolic arterial pressure (DAP), PaCO_2_and SaO_2_. **A** RI and age: the relationship is significant for total population R = 0.574 (p < 0.0001*), for normal glomerular filtration rates (GFRs) (p = 0.0246*) and for decreased GFRs (p = 0.0201*) but not observed for augmented renal clearance (ARC) (p = 0.0639). **B** RI and measured CL_CR_: relationship is significant for total population R = − 0.424 (p = 0.0005*), for normal GFRs (p = 0.0399*) and for decreased GFRs (p = 0.0439*) but not observed for ARC (p = 0.4198). **C** RI and DAP: the relationship is significant for the total population R = − 0.368 (p = 0.0028*), normal GFRs (p = 0.0061*) but not observed for decreased GFRs (p = 0.3794) and for ARC (p = 0.9730). **D** RI and PCO2: the relationship is significant for the overall population R = 0.270 (p = 0.0310*) **E** RI and SaO2: the relationship is significant for the overall population R = − 0.284 (p = 0.0231*). *Statistically significant

A significant positive correlation was observed between the RI and age, urea (urea: R = 0.419, p < 0.0016), and PaCO_2_. Conversely, a significant negative correlation was found between the RI and measured creatinine clearance, diastolic arterial pressure (DAP), and SaO_2_ (Fig. 1).

Multivariate analysis using multiple regressions was conducted, considering the integration or non-integration of measured creatinine clearance (CrCl) depending on whether the RI was regarded as a consequence or an indicator of glomerular filtration rate (GFR). In both models, it was found that the only significant factors influencing the RI level were age (p < 0.0001), PaCO2 (p = 0.0056), and DAP (p = 0.0416).

Table 2 provides an overview of the relationship between the RI, clinical data, and biological data for the three groups classified based on GFR levels (kidney failure, normal GFR, and ARC).

**Table 2:** Clinical, biological and hemodynamic data in each of the groups characterized by their level of GFRs

	Normal GFR n = 35		ARC n = 17		Kidney failure n = 12		p
	Median	CI 95%	Median	CI 95%	Median	CI 95%	
Age (years)	60	52–68	28	22–48	77.5	57–84.5	0.00001*
Albuminemia (g/L)	24	18.6–27	23	19.5–27	17.6	NA	0.548187
BMI (kg/m^2^)	25	24–27	25	23–28	28	24.4–31	0.19521
SAPS 2	55	43–64	48	32–53	47	42–64.9	0.34085
SOFA Score	5.5	4–8	6	4–8	4	2.2–4	0.08673
Delay between ICU admission and inclusion (days)	4	3–6.5	4	3–6	12	4–15	0.08731
PEEP (cmH_2_O)	6	5–7	6	5–8	6	0–8	0.71223
Plasma creatinine (µmol/L)	58	49–69	56	47–64	141	76–182	0.00004*
BUN (mmol/L)	6.2	5.3–8.2	5.4	3.5–7	17.7	7.2–28	0.00177*
CKD-EPI (mL/min/1.73 m^2^)	104.56	95.9–111	127.4	122.6–137.4	39.98	25–94.3	< 0.000001*
Measured creatinine clearance (mL/min/1.73 m^2^)	115.83	105–126	204	160.3–265.2	33	23–43.5	< 0.000001*
pH	7.43	7.42–7.45	7.43	7.41–7.46	7.4	7.34–7.47	0.64280
PaO2 (mmHg)	82.2	71–102	85.7	71–111	81.6	69.8–108.3	0.89991
SaO2 (%)	96.4	95–98	97.8	95–98.5	97.2	94–99	0.94325
PaCO2 (mmHg)	37.2	35.9–40	39.4	32.2–41.2	35.9	32.6–45.8	0.71234
Plasma total CO2 content (mmol/L)	25.5	24.9–27	25.6	24.4–27	24	20.4–26.4	0.41107
Hemoglobin (g/dL)	10.1	9.5–10.5	10.2	9.3–11.4	10.2	8.9–12.4	0.90643
SAP (mmHg)	127	119–134	138	130–149	130	122–152	0.09584
DAP (mmHg)	64	58–68	71	64–81	62	50–74	0.08861
MAP (mmHg)	84	80–87.8	90	85–95	84	72–94	0.08589
Pulse pressure (mmHg)	65	56.5–68	66	53–84	69.5	59.9–84	0.42530
Heart rate (bpm)	88	84–94	90	80–112	86	76–102	0.76239
Resistivity Index	0.71	0.66–0.73	0.62	0.596–0.69	0.76	0.69–0.81	0.00533*
Cause of admission							0.0001*
Polytrauma	6 (17.1%)		12 (70.6%))		0 (0%)		
Surgical	10 (28.6%)		4 (23.5%)		4 (33.3%)		
Medical	19 (54.3%)		1 (5.9%)		8 (66.7%)		
Sex F/M	13 (37.1%) / 22 (62.9%)		3 (17.6%) / 14 (82.4%)		3 (25%) / 9 (75%)		0.3265

Normal GFR (glomerular filtration rate): measured CL_CR_ between 60 and 150 mL/min/1.73 m^2^ (for female) and 160 mL/min/1.73 m^2^ (for male)

ARC (Augmented Renal Clearance): measured CL_CR_ > 150 (for female) et 160 (for male) mL/min/1.73 m^2^

Kidney failure: Measured CL_CR_ < 60 mL/min/1.73 m^2^

*BMI* body mass index, *BUN* blood urea nitrogen, *CI* confidence interval, *DAP* diastolic arterial pressure, *ICU* intensive care unit, *MAP* mean arterial pressure, *NA* not applicable, *PEEP* positive end-expiratory pressure, *SAP* systolic arterial pressure, *SAPS* Simplified Acute Physiology Score, *SOFA score* Sepsis-related Organ Failure Assessment score

*Statistically significant

**Fig. 2:**
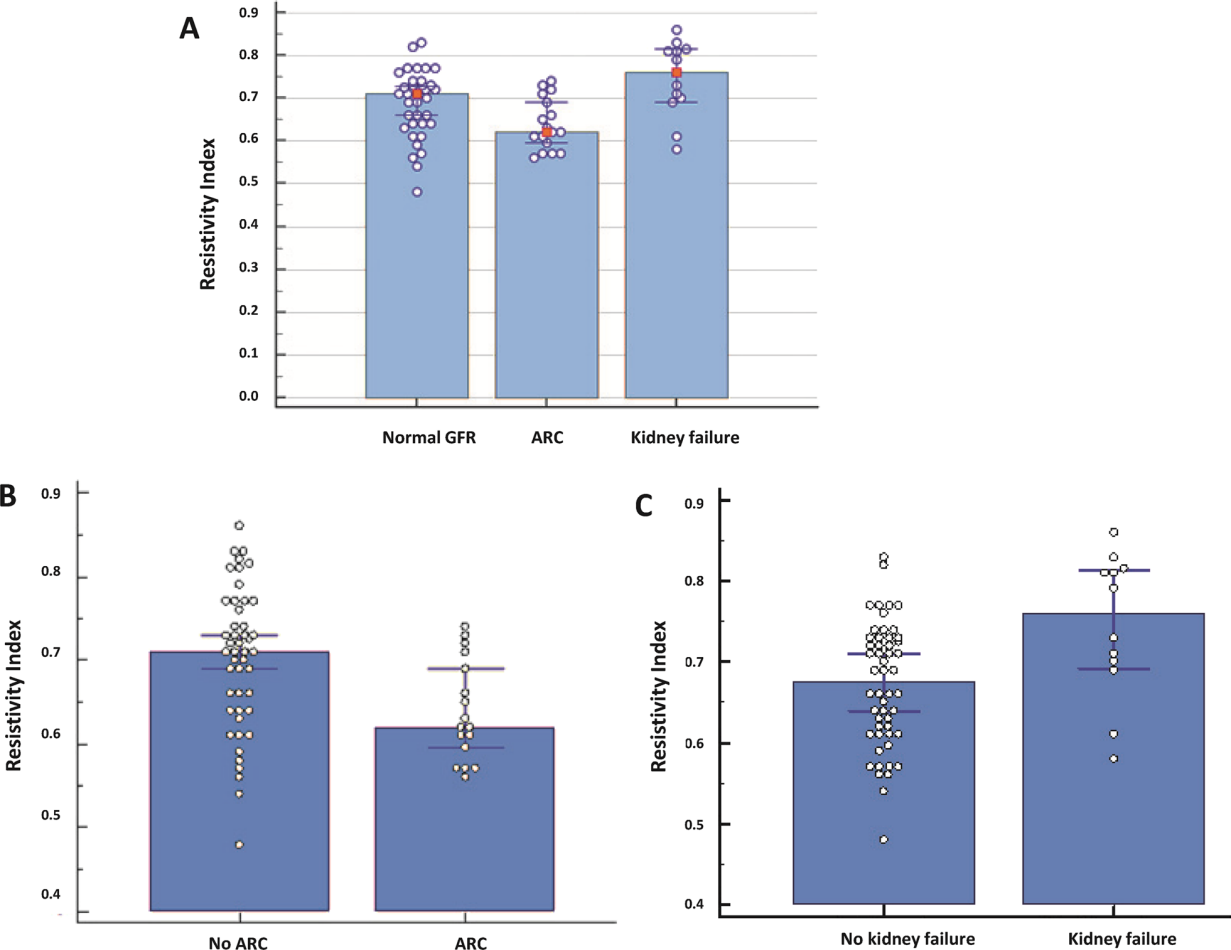
Comparison of Resistivity Index (RI) of patients according to their glomerular filtration rate (GFR) level. **A** RI in patients with normal GFR, ARC and decreased GFR. **B** RI of patients with ARC vs. non ARC. **C** RI of patients with decreased DFG vs. normal GFR or ARC patients

Figure 2 illustrates the RI levels in different groups based on their glomerular filtration rate (GFR). Among the 47 patients without augmented renal clearance (non-ARC), the RI was measured at 0.71 (0.69–0.73), whereas in the 17 patients with ARC, it was 0.62 (0.596–0.69) (p = 0.0059) (Fig. 2B). The difference primarily stemmed from the patients with kidney failure (n = 12), who exhibited an RI of 0.76 (0.69–0.81) compared to 0.68 (0.64–0.71) (p = 0.0156) (Fig. 2C).

The ROC curve analysis determined an RI threshold of 0.77 (gray zone: 0.66–0.77) for detecting kidney function impairment. Notably, the discrimination ability was relatively low, with an AUC (= 0.73) below the threshold of 0.8. Considering the limited sample size of patients with kidney failure and the wide “gray zone” of RI values within this population, we selected an RI threshold of 0.7 (previously used in the literature with a larger patient cohort for screening kidney function impairment). This threshold falls within the predefined gray zone. The results are presented in Table 3.

**Table 3:** Comparison of clinical, biological and hemodynamic data in patients with Resistivity Index (RI) > 0.7 vs. RI ≤ 0.7

	RI ≤ 0.7					RI ≥ 0.7					p
	N	Median	CI 95%	Minimal	Maximal	N	Median	CI 95%	Minimal	Maximal	
Resistivity Index	34	0.62	0.61–0.64	0.48	0.70	30	0.74	0.73–0.77	0.71	0.86	< 0.0001*
Age (years)	34	48	29.8–52	17	85	30	69	61–75	23	87	< 0.0001*
BMI (kg/m^2^)	34	25	23–28	17	37	30	25	24–28	18	35	0.6092
SAPS 2	34	48.5	43–53	23	86	30	53	42–68	24	91	0.4004
SOFA Score	32	6	4–8	1	11	30	4	3–6	1	12	0.0522
Albuminemia (g/L)	26	23	20–26	14	31	11	24	16.8–26	15	36	0.8546
Measured creatinine clearance (mL/min/1.73 m^2^)	34	136.6	121–160	36.7	296	30	85.3	61.9–108	9	319	0.0005*
BUN (mmol/L)	25	5.7	3.7–7.3	1.9	25.3	29	7.4	5.9–10.3	3.7	52	0.0241*
Heart rate (bpm)	34	95	86–105	39	150	29	86	80–91	68	102	0.0201*
SAP (mmHg)	34	135	126–140	101	175	30	128	123–134	90	177	0.2878
DAP (mmHg)	34	68.5	65–77	50	98	30	58	55–66	42	87	0.0019*
MAP (mmHg)	34	87	84–94	68	120	30	82	75–87	59	114	0.0092*
Pulse pressure (mmHg)	34	65	56–69	29	97	30	66	60–74	45	106	0.2417
pH	34	7.43	7.41–7.45	7.32	7.52	30	7.43	7.4–7.48	7.24	7.55	0.5050
PaO_2_ (mmHg)	34	85.7	76–110	62	177	30	79.7	70–93	41	191	0.0966
PaCO_2_ (mmHg)	34	36.6	33-39.5	29	54	30	39.75	36–44	27	66.5	0.0769
Plasma total CO_2_ content	34	25.3	24–26	18.3	38.2	30	25.5	25–28	17	41	0.4273
SaO_2_ (%)	34	98	96–98	91	99	30	96	94–97	82	100	0.1301
Hemoglobin (g/dL)	34	10.4	9.9–11.2	7.8	13.6	30	9.7	9-10.5	8	13	0.1186
PEEP (cmH_2_0)	33	6	5–7	0	10	29	6	5–8	0	12	0.5621
Cause of admission											0.0098*
Polytrauma	15 (44.1%)					3 (10%)					
Surgical	7 (20.6%)					11 (36.7%)					
Medical	12 (35.3%)					16 (53.3%)					

*BMI* body mass index, *BUN* blood urea nitrogen, *CI* confidence interval, *DAP* diastolic arterial pressure, *ICU* intensive care unit, *MAP* mean arterial pressure, *PEEP* positive end-expiratory pressure, *SAP* systolic arterial pressure, *SAPS* Simplified Acute Physiology Score, *SOFA score* Sepsis-related Organ Failure Assessment score

*Statistically significant

**Fig. 3:**
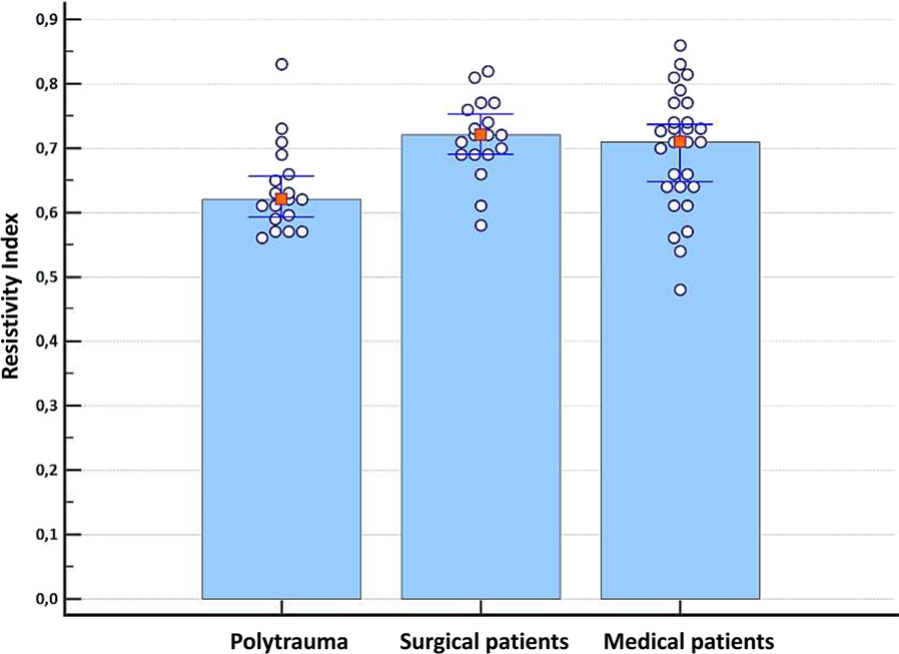
Comparison of RI of polytrauma patients, patients hospitalized for surgical reason or medical reason

The most discriminatory variable appears to be age, with a threshold of > 58 years, demonstrating a sensitivity of 77%, specificity of 85%, positive predictive value (PPV) of 82%, negative predictive value (NPV) of 81%, and an area under the receiver operating characteristic (AUROC) curve of 0.82. However, for the other variables, the ROC curve analysis did not yield a statistically significant threshold (AUC not different from 0.5).

Furthermore, the RI exhibits variations based on the underlying condition necessitating admission to the intensive care unit, as depicted in Fig. 3.

The RI values differ among patient groups, with a measurement of 0.62 (0.59–0.66) in multiple trauma patients, 0.72 (0.69–0.75) in postoperative patients, and 0.71 (0.65–0.74) in medical patients (p = 0.00504). The significant difference observed is primarily attributed to the multiple trauma group.

### Multivariate analysis

The multivariate analysis of factors associated with an RI > 0.7, incorporating both continuous and categorical variables, is presented in Table 4.

**Table 4:** Multivariate analysis of factors explaining a Resistivity Index (RI) > 0.7 (excluding measured measured creatinine clearance which by definition is related to a RI > 0.7)

RI > 0.7	p	OR	CI 95%
Age > 58 years	0.0003*	**18.672**	3.859–90.329
PaCO_2_ (mmHg)	0.0267*	**1.132**	1.015–1.264
DAP (mmHg)	0.2053	0.957	0.894–1.024
AUC	0.89 [0.79–0.96]		
Test Hosmer Lemeshow	0.84		
Percentage of cases correctly classified	82.8%		

*AUC* area under the curve, *CI* confidence interval, *DAP* diastolic arterial pressure, *OR* odd ratio

*Statistically significant

However, multiple trauma was not included in the model due to its strong correlation with age. It was observed that 96% of multiple trauma patients were under the age of 58, and this variable lost significance when age was included in the analysis.

The influence of age and PaCO2 on the RI level was confirmed. The RI tends to increase after the age of 58, and it also increases with higher levels of PaCO_2_, although a specific threshold for PaCO_2_ could not be determined.

**Fig. 4:**
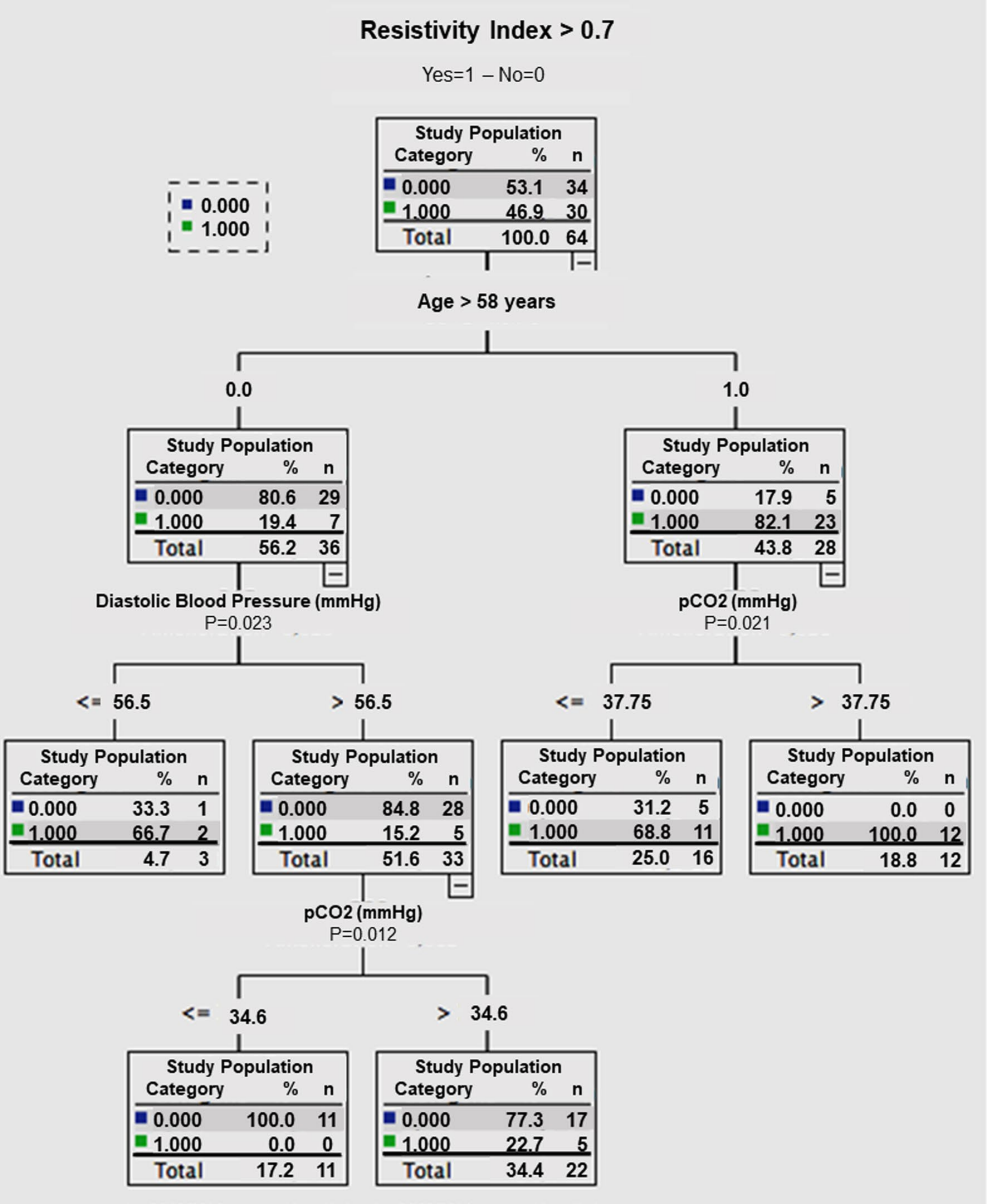
Partitioning of patients according to their Resistivity Index (RI) > or ≤ 0.7 according to their age (> or ≤ 58 years), their PaCO_2_ level and their Diastolic Arterial Pressure (DAP).

The Classification and Regression Tree (CART) analysis was employed to define several thresholds based on the data. Figure 4 illustrates the distribution of individuals considering these four factors, with a predictive accuracy of 82.8%, matching that of the logistic regression model. The CART analysis helped establish PaCO_2_ thresholds within different subgroups, along with a threshold for diastolic arterial pressure (DAP) for patients under the age of 58.

In summary, a patient above the age of 58 with a PaCO_2_ > 37.75 mmHg has a 100% likelihood of having an RI > 0.7. On the other hand, a patient under the age of 58 with a DAP > 56.5 mmHg and a PCO_2_ < 34.6 mmHg has a 100% chance of having an RI < 0.7.

## Discussion

Our study highlights that in the ICU setting, the interpretation of the RI should be based on age and PaCO_2_ thresholds. The RI serves as a reproducible and user-friendly tool for early screening and prognostication of AKI in the ICU [21]. However, there are limitations to performing this examination due to specific circumstances faced by ICU patients, such as mechanical ventilation, postoperative abdominal surgery, and abdominal distension [23, 24]. Additionally, when interpreting the RI, it is important to consider other non-renal confounding factors, including systemic atherosclerosis that alters renal vascular compliance, the overall hemodynamic status, and intra-abdominal pressure [8, 10, 13, 20, 25–29].

Our study stands out due to its focus on examining the influence of arterial blood gases, particularly PaCO_2_, on the RI in ICU patients under routine care conditions. Previous studies have suggested that severe hypoxemia in rats or patients on mechanical ventilation leads to renal vasodilation, while significant increases in PaCO_2_ result in vasoconstriction [17, 30]. However, the findings from these studies were limited in their applicability to clinical practice due to small sample sizes and experimental conditions [16, 17]. The strength of our study lies in the inclusion of a larger population (64 patients) and its execution in real-world clinical conditions, regardless of the admission reason or patient history. Moreover, simultaneous laboratory and Doppler procedures were performed, allowing for the application of these results to everyday ICU settings. However, despite the larger sample size, we were unable to establish a significant relationship between PaO_2_ (or SaO_2_) and variation in RI, which contradicts findings from other studies conducted on patients undergoing postoperative cardiac surgery or those with acute lung injury (ALI) [17, 31].

We selected an RI threshold of 0.7 based on relevant literature, as it has been demonstrated to be associated with the occurrence of organic acute kidney failure [12, 32]. An RI value below 0.7 is indicative of reversible acute functional kidney failure without renal parenchymal lesions [12]. Additionally, previous studies have shown that ICU patients admitted for septic shock with an RI > 0.74 have an elevated risk of developing kidney dysfunction within the first five days of admission, with a sensitivity of 78% and a specificity of 77% [8].

Previous studies have not been able to establish a direct correlation between the resistive index (RI) and renal blood flow or glomerular filtration rate (GFR) [30, 33, 34]. However, in hypoxemic and hypercapnic patients with severe chronic obstructive pulmonary disease (COPD), the pulsatility index (PI), which is conceptually similar to the RI, decreased after exposure to hyperoxia. Conversely, the PI increased upon administration of inhaled CO2, indicating an elevation in renal vascular resistance and a decrease in renal blood flow [16]. Similarly, in our study, PaCO_2_ significantly influenced the RI (p = 0.0416) across patients with augmented renal clearance (ARC), normal GFR, or glomerular hypofiltration. In each of these groups, both PaCO_2_ and RI increased concurrently. The multivariate analysis further confirmed the impact of PaCO_2_ on the RI (odds ratio 1.132, 95% CI: 1.015–1.264, p = 0.0267). Through multivariate analysis using classification and regression tree, we found that ICU patients aged over 58 years with a PaCO_2_ level exceeding 37.75 had a 100% risk of having an RI above 0.7. In certain conditions such as acute respiratory distress syndrome (ARDS), higher thresholds of PaCO_2_ are generally tolerated. Nevertheless, based on the results of our study, the actual effects of hypercapnia on renal parenchyma and its function still require further evaluation.

As evidenced in existing literature, age has a direct influence on the glomerular filtration rate (GFR), which was reduced in approximately 20% of the patients in our study (median age of patients with kidney failure: 77.5 years, 95% CI: 57–84.5). Additionally, there is a corresponding increase in the resistive index (RI) with age [35]. This rise can be attributed to increased arterial rigidity and decreased compliance [36]. Our study found comparable results. Our study yielded similar findings, confirming this relationship between age and RI.

We also identified a correlation between diastolic arterial pressure (DAP) and the resistive index (RI). Previous studies have explored the relationship between global hemodynamics and the RI, indicating that an increase in mean arterial pressure (MAP) due to noradrenaline administration leads to an elevated RI with a threshold effect [10]. This suggests that renal Doppler measurements could aid in determining the optimal MAP for kidney tissue perfusion in individual patients and serve as a valuable tool for titrating hemodynamic treatment in septic shock. However, it is important to note that this global hemodynamic effect likely has a moderate impact on renal hemodynamics, considering the numerous independent factors involved in regulating renal blood flow. The limitation of this relationship is evident in our study, as the multivariate analysis did not establish a connection between macro hemodynamics and the RI across all observed patient groups. The influence of age, arterial hypertension, and other factors related to arterial stiffness have been extensively demonstrated to affect the RI and are likely confounding factors in the observed association between DAP and RI in our univariate analysis.

The resistive index (RI) has demonstrated its predictive value in determining the reversibility of acute kidney failure and its potential for guiding hemodynamic optimization, especially in cases of septic shock. However, its diagnostic performance cannot be utilized as a screening tool for augmented renal clearance (ARC) [37]. Although RI levels show statistically significant differences between ARC and non-ARC patients (RI of 0.62 (0.596–0.69) vs. 0.71 (0.69–0.73), respectively; p = 0.0059), this disparity is influenced by the presence of patients with renal failure in the non-ARC group, artificially inflating the RI of that group. Therefore, a dedicated study with a larger sample size is necessary to explore the potential correlation between the RI and ARC more comprehensively.

Our study has several limitations. One key limitation is the complexity of interpreting the resistive index (RI) due to the influence of multiple factors, including mean arterial pressure, pulse pressure, vascular compliance, oxygenation levels, and intra-abdominal pressure [8, 16, 17, 25–29, 38]. Among these confounding factors, some may yield conflicting conclusions, thereby reducing the diagnostic performance of this tool [38]. Our study specifically focused on the influence of arterial blood gases on the RI, allowing for a detailed analysis of this aspect. Our findings emphasize the importance of considering normalized PaCO2 levels and establishing age and diastolic arterial pressure (DAP) thresholds, which should be investigated in future studies to enhance the interpretation of the RI.

## Conclusion

The resistive index (RI) serves as a noninvasive, fast, and reliable diagnostic tool that can help predict the reversibility of acute kidney injury (AKI) and tailor individualized hemodynamic management for patients. Our study demonstrates the positive influence of PaCO_2_ on the RI, particularly in hypercapnic patients. It highlights the importance of analyzing the RI alongside normalized PaCO_2_ levels and age and diastolic arterial pressure (DAP) thresholds. Further research is warranted to develop a scoring system that integrates these cofactors, enabling more reliable interpretation of the RI results.

## Data Availability

The datasets used and/or analysed during the current study are available from the corresponding author on reasonable request.
